# On the generation of the MSD-Ѱ class of defective HIV proviruses

**DOI:** 10.1186/s12977-019-0481-2

**Published:** 2019-07-11

**Authors:** Atze T. Das, Alexander O. Pasternak, Ben Berkhout

**Affiliations:** 1 Laboratory of Experimental Virology, Department of Medical Microbiology, Amsterdam University Medical Centers, University of Amsterdam, Amsterdam, The Netherlands; 2 Laboratory of Molecular Virology, Department of Life and Environmental Sciences, University of Cagliari, Cagliari, Italy

**Keywords:** HIV, Reservoir, Defective genomes, Splicing, Polyadenylation

## Abstract

Antiretroviral therapy (ART) can effectively suppress ongoing HIV replication and block disease progression, but the infection is never cured due to the persistence of a small pool of latently infected cells hosting integrated replication-competent HIV proviruses. However, the vast majority of HIV proviruses in ART-treated patients are replication-incompetent due to a variety of genetic defects. Most defective proviruses (around 90%) contain large internal deletions or are G-to-A hypermutated, resulting in destruction of most if not all viral open reading frames, which is consistent with the idea that cytotoxic T cells (CTLs) effectively remove cells that produce viral antigens. An intriguing subclass of defective proviruses (around 10%) that are consistently detected in such patients carry a small deletion or a point mutation in a relatively precise and well conserved region near the 5ʹ end of the HIV genome, in the area that encodes the major splice donor (MSD) site and the packaging signal Ѱ in the viral RNA genome. Why this subclass of proviruses is defective has never been properly understood. We now propose a mechanistic scenario for how these MSD-Ѱ mutations can prevent viral protein expression. Based on ample results in literature, we argue that MSD inactivation triggers the activity of the 5ʹ-polyadenylation site, resulting in the production of ultra-short non-protein-coding HIV transcripts.

Defective HIV proviruses are produced in large quantities during natural infection due to mutations introduced during the error-prone process of HIV reverse transcription and APOBEC-induced hypermutation [1, 2]. In untreated patients, this process is counterweighted by the unhindered production of new intact proviruses by virus replication, but in patients on suppressive antiretroviral therapy (ART), defective proviruses accumulate to very high levels [3]. Bruner et al. [4] showed that even in patients who started ART during early infection, 93% of all proviruses were defective, and if HIV replication was blocked by ART during chronic infection, this percentage of defective HIV genomes reached 98%. Similar percentages of defective proviruses have been reported by other groups [5, 6]. It is thought that ART selects for defective proviruses due to continuous cytotoxic T cell (CTL)-mediated surveillance for cells that produce foreign viral antigens, which in ART-treated patients is not counterweighted by virus replication [7, 8]. CTL pressure does decrease after initiation of ART due to decreased antigen exposure, but does not disappear completely [9].

Although defective HIV proviruses are considered by many clinically irrelevant, they do frustrate the accurate measurement of the clinically relevant reservoir of intact HIV genomes that forms a major barrier to curing infected individuals. Furthermore, defective proviruses can be expressed and recognized by the host immune system, which may “distract” CTLs from eliminating the latent reservoir [7, 8, 10] and contribute to the increased levels of immune activation and inflammation on ART [11, 12]. It is therefore important to analyse the pool of defective HIV genomes in greater detail [13].

The structure of most of these defective HIV genomes does confirm the requirement of little or no viral protein expression as the HIV open reading frames acquire inactivating mutations, either by means of large deletions or hypermutation (Fig. 1). A detailed molecular analysis confirmed the protein expression defect for these proviruses [3]. However, a significant subclass of defective HIV genomes is explained less easily. This distinct MSD-Ѱ subclass carries a relatively small deletion in the non-coding part of the HIV genome between the LTR promoter and the first Gag open reading frame (Fig. 1). This region encodes the 5ʹ-untranslated region (5ʹ-UTR) of the HIV RNA genome and contains many post-transcriptional replication signals, including the major splice donor (MSD) that is used in the generation of all spliced HIV transcripts and the packaging signal Ѱ that ensures the selective encapsidation of HIV RNA in assembling virion particles [14]. The magnitude of this MSD-Ѱ class of defective HIV-1 proviruses varies somewhat between studies, ranging from 5 and 6.5% in early studies [3, 4] to 11% in a recent study using a novel provirus sequencing assay [5].

**Fig. 1:**
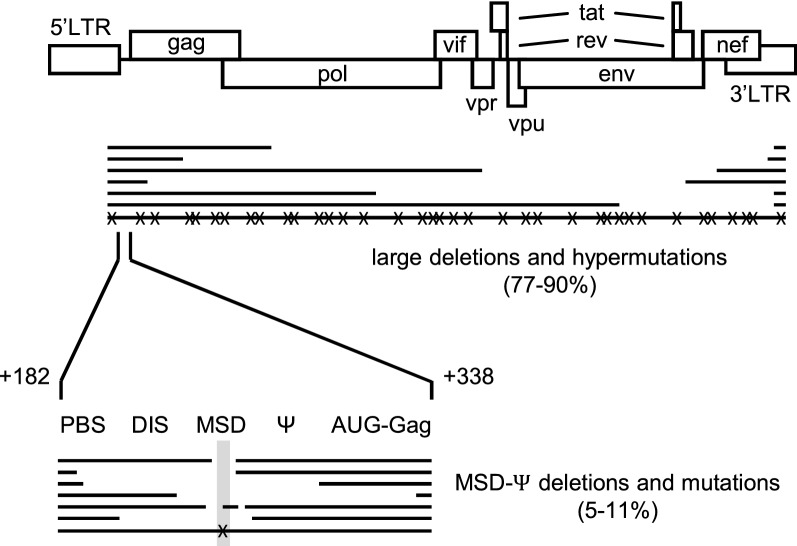
Schematic of the mutations observed in defective HIV proviruses. The HIV genome is depicted on top, with underneath the large deletions and hypermutations (X, nucleotide substitution) that are found in 77–90% of defective proviruses in patients receiving therapy [3, 5, 13]. The bottom part represents a blow-up of the untranslated leader region of the HIV genome (RNA coordinates + 182/+ 338) that is affected in the MSD-Ѱ class of defective proviruses. We marked the position of several replication signals (*PBS* primer binding site, *DIS* dimerization initiation signal, AUG-Gag is the first start codon that is used for Gag translation). The deletions and mutations reported by Ho et al. [3] are schematically depicted, showing clustering around the MSD (shadowed)

The persistence of the MSD-Ѱ mutated proviruses during ART suggests an inability to produce viral proteins, but no explanation for such a production defect was yet presented. In fact, MSD inactivation was shown to induce alternative RNA splicing events that can give rise to the expression of viral proteins, e.g. Tat and Rev, or aberrant proteins [7]. Although the level of gene expression can be reduced for these HIV genomes, e.g. due to reduced Tat levels, the corresponding host cells will still be recognized and cleared by CTLs. This MSD-Ѱ class thus far could not be fully understood. Based on extensive literature findings in the field of HIV molecular biology that thus far were ignored, we report an attractive, yet simple explanation for the protein production defect of MSD-Ѱ mutated HIV genomes.

We started by a sequence alignment of the previously reported MSD-Ѱ mutants to identify the critical motifs that were consistently affected. For instance, Fig. 1 shows the deletions reported in the study by Ho et al. [3]. All deletions include the MSD motif, whereas Ѱ sequences do frequently remain present, arguing for a functional role of the MSD motif that controls HIV-1 RNA splicing. In addition, also proviruses with point mutations were reported in the MSD region, e.g. affecting the critical intronic GU dinucleotide of the splice donor site (UG-GU mutated to UG-G**G**) [3]. Importantly, it was demonstrated that such a point mutant can exhibit a severe replication defect in reconstructed viruses [3].

The literature on HIV molecular biology does provide clues on the MSD-Ѱ mystery. Previous work indicated that the process of HIV RNA polyadenylation is highly regulated. The biological challenge is that the viral RNA genome encodes two identical polyadenylation (polyA or pA) signals as part of the 5ʹ and 3ʹR (repeat) regions near the 5ʹ and 3ʹ ends of HIV RNA. Therefore, regulation is of key importance to suppress the 5ʹ pA site and/or to selectively activate the 3ʹ pA site (Fig. 2a). Work from several groups proposed multiple-layer regulatory mechanisms to achieve negligible 5ʹ pA activity and full 3ʹ pA activity. An early model indicated that the 5ʹ pA site is not frequently used because of its close proximity to the promoter, suggesting that the transcriptional complex needs to mature to become sensitive to pA signals [15]. We demonstrated that both sites are partially suppressed by being part of a local hairpin that reduces binding of cleavage polyadenylation specificity factor (CPSF) [16, 17]. Complete inactivity of the 5ʹ pA site was demonstrated to be linked to the MSD site positioned about 200 nucleotides downstream [18]. Importantly, these results were obtained with HIV proviral constructs, as such emphasizing the physiological importance of this MSD-pA interaction. Efficient interaction of U1 snRNP with the MSD was reported to be critical for complete inactivation of the 5ʹ pA site [19] and follow-up work indicated an important role for the stem-loop 1 of the U1 snRNP [20]. Novel mutational approaches recently confirmed the importance of the MSD region for HIV gene expression [21] and the role of the MSD in regulated polyadenylation [22]. No MSD is present downstream of the 3ʹ pA site, thus avoiding its inactivation. To complete the regulatory mechanism, the 3ʹ pA is also partially suppressed by local RNA structure but able to gain full activity due to an upstream splicing enhancer (USE) element that is uniquely present upstream of this site. This enhancer was shown to act as CPSF entry site for the structurally obstructed 3ʹ pA site [23, 24]. Figure 2a illustrates this complex regulatory mechanism, which seems unique for HIV among the *Retroviridae*.

**Fig. 2:**
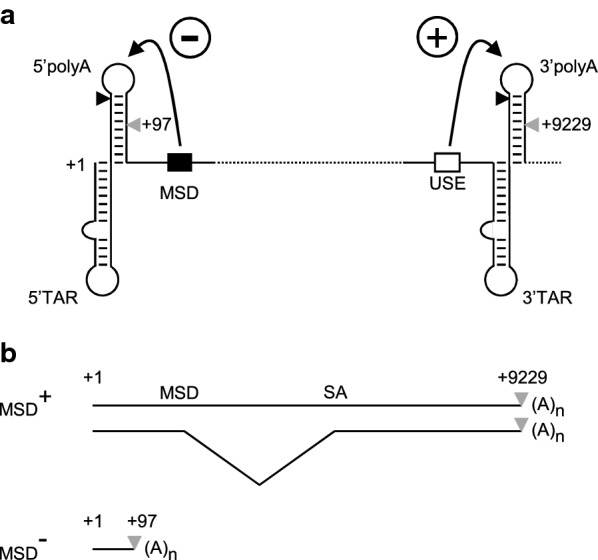
Model for 5ʹ pA site activation in the HIV genome by MSD-inactivation. **a** Cartoon of the proposed model for pA site regulation in the HIV RNA genome: suppression of the 5ʹ pA site by the downstream MSD and activation of the 3ʹ pA site by the upstream USE. See the text for further details. The pA hairpins and the upstream TAR hairpins are shown. The pA hairpin structure suppresses both 5ʹ and 3ʹ polyadenylation and allows the MSD/USE control. The 3ʹTAR hairpin juxtaposes the USE and the 3 pA site, which may enhance USE-mediated activation of polyadenylation [14]. The black triangles indicate the position of the AAUAAA polyadenylation signal. The grey arrow represents the actual site of polyadenylation at position 97 (5ʹ copy) or 9229 (3ʹ copy). **b** Illustrated are the HIV transcripts expected for wild-type MSD^+^ viruses (full-length unspliced and spliced versions, SA is one of the many splice acceptors in the HIV genome) and mutant MSD^−^ proviruses (only short TAR transcripts). (A)n is the polyA tail

With this mechanistic background, it can easily be understood that inactivation of MSD by mutation or deletion can trigger an effective shutdown of HIV transcription through activation of the 5ʹ pA site. Thus, it follows that a small characteristic HIV transcript of 97 nucleotides plus polyA tail will be synthesized in the cells that carry a MSD-mutated provirus, as illustrated in Fig. 2b. This non-coding HIV transcript that encompasses the TAR motif was indeed reported by the Proudfoot laboratory back in 1995 [18]. This short TAR transcript is polyadenylated at the 5ʹ-pA site and was confirmed in studies on the regulatory role of the polyA hairpin structure [25–27]. As this short transcript encodes the complete TAR element it may be processed into the TAR miRNA, of which the precise role has not been determined yet [28, 29].

Although removal of all HIV coding capacity by a large internal deletion is also very effective in preventing HIV gene expression, MSD inactivation is arguably the most elegant way to produce non-expressing proviruses, the host cells of which will survive under massive CTL pressure. This 5ʹ pA activation model seems much more relevant to explain the loss of HIV protein production than the proposed model of alternative usage of splice sites, which at best could reduce and not interrupt HIV protein expression. Although the inactivation of HIV splice sites can indeed trigger the usage of new splice sites [30–33], this does not prevent protein translation and consequently CTL recognition.

Consistent with the mechanistic model presented in Fig. 2a, short TAR-containing HIV transcripts are produced in treated patients at a level at least 10-fold higher than extended HIV transcripts [34, 35]. These authors did assume that short TAR-containing transcripts represent abortive transcripts. Our model predicts that short transcripts that are polyadenylated at the 5ʹ-pA site may significantly contribute to this small RNA pool.

One could argue that the same end result, that is activation of the 5ʹ-pA site, could be achieved by weakening or opening of the local polyA hairpin structure that suppresses CPSF binding [17, 25]. However, this would require surgical precision for the provirus mutation as the sequence elements that control the polyadenylation process should not be affected. These include the canonical AAUAAA signal and the actual cleavage site that are embedded in the polyA hairpin [24, 36]. This may explain why 5ʹ pA-activation by hairpin destabilization is not observed, at least not frequently.

The 5ʹ pA activation model does not only apply to the relatively minor MSD-Ѱ class of defective HIV proviruses, it will also relate to those members of the two major classes of defective proviruses with large deletions or hypermutated genomes in which the MSD is destroyed. The latter two classes were supposed to be defective by inactivation of one or multiple open reading frames, but 5ʹ pA activation provides a dominant mechanism to abort any viral protein expression. This new mechanism may therefore also be very relevant for scenario’s dealing with the relevance of ongoing viral protein expression [6, 7].

The generation of variant HIV genomes is the result of two independent processes: mutation and subsequent selection of the most fit virus. In this case, host cells carrying a HIV provirus with a protein production defect will survive preferentially under intense CTL pressure that has been built in infected individuals during months or years of unsuppressed virus replication. The mechanistic MSD-pA scenario that we propose suggests that the cells with proviruses carrying MSD-inactivating mutations are selected because of their non-protein-expressing phenotype. Although not likely to be of decisive influence, the presence of hotspots of viral recombination may also influence the type of MSD deletions that occur (Fig. 1). In particular, this MSD-Ѱ part of the viral RNA genome is highly structured and can cause the viral Reverse Transcriptase to pause [37], which can induce recombination and MSD deletion. In any case, the subsequent selection of cells that do not express viral proteins is the key event.

A complete understanding of the pool of defective HIV proviruses remains of critical importance for accurate measurement of the latent virus reservoir. There may be multiple ways to inactivate HIV and we here describe that—besides prominent deletions and hypermutations—more subtle changes like MSD mutations can also destroy HIV expression.

## Data Availability

Not relevant.

## Abbreviations

ART: antiretroviral therapy
CTL: cytotoxic T lymphocyte
MSD: major splice donor
5ʹ-UTR: 5ʹ-untranslated region
polyA or pA: polyadenylation
CPSF: cleavage and polyadenylation specificity factor
USE: upstream splicing enhancer

## References

[1] Abram ME, Ferris AL, Shao W, Alvord WG, Hughes SH (2010). Nature, position, and frequency of mutations made in a single cycle of HIV-1 replication. J Virol.

[2] Harris RS, Bishop KN, Sheehy AM, Craig HM, Petersen-Mahrt SK, Watt IN, Neuberger MS, Malim MH (2003). DNA deamination mediates innate immunity to retroviral infection. Cell.

[3] Ho YC, Shan L, Hosmane NN, Wang J, Laskey SB, Rosenbloom DI, Lai J, Blankson JN, Siliciano JD, Siliciano RF (2013). Replication-competent noninduced proviruses in the latent reservoir increase barrier to HIV-1 cure. Cell.

[4] Bruner KM, Murray AJ, Pollack RA, Soliman MG, Laskey SB, Capoferri AA, Lai J, Strain MC, Lada SM, Hoh R (2016). Defective proviruses rapidly accumulate during acute HIV-1 infection. Nat Med.

[5] Hiener B, Horsburgh BA, Eden JS, Barton K, Schlub TE, Lee E, von Stockenstrom S, Odevall L, Milush JM, Liegler T (2017). Identification of genetically intact HIV-1 proviruses in specific CD4(+) T cells from effectively treated participants. Cell Rep.

[6] Pinzone MR, VanBelzen DJ, Weissman S, Bertuccio MP, Cannon L, Venanzi-Rullo E, Migueles S, Jones RB, Mota T, Joseph SB (2019). Longitudinal HIV sequencing reveals reservoir expression leading to decay which is obscured by clonal expansion. Nat Commun.

[7] Pollack RA, Jones RB, Pertea M, Bruner KM, Martin AR, Thomas AS, Capoferri AA, Beg SA, Huang SH, Karandish S (2017). Defective HIV-1 proviruses are expressed and can be recognized by cytotoxic T lymphocytes, which shape the proviral landscape. Cell Host Microbe.

[8] Imamichi H, Dewar RL, Adelsberger JW, Rehm CA, O’Doherty U, Paxinos EE, Fauci AS, Lane HC (2016). Defective HIV-1 proviruses produce novel protein-coding RNA species in HIV-infected patients on combination antiretroviral therapy. Proc Natl Acad Sci USA.

[9] Cartwright EK, Spicer L, Smith SA, Lee D, Fast R, Paganini S, Lawson BO, Nega M, Easley K, Schmitz JE (2016). CD8(+) lymphocytes are required for maintaining viral suppression in SIV-infected macaques treated with short-term antiretroviral therapy. Immunity.

[10] Huang SH, Ren Y, Thomas AS, Chan D, Mueller S, Ward AR, Patel S, Bollard CM, Cruz CR, Karandish S (2018). Latent HIV reservoirs exhibit inherent resistance to elimination by CD8+ T cells. J Clin Invest.

[11] Hatano H, Jain V, Hunt PW, Lee TH, Sinclair E, Do TD, Hoh R, Martin JN, McCune JM, Hecht F (2013). Cell-based measures of viral persistence are associated with immune activation and programmed cell death protein 1 (PD-1)-expressing CD4+ T cells. J Infect Dis.

[12] Deeks SG, Tracy R, Douek DC (2013). Systemic effects of inflammation on health during chronic HIV infection. Immunity.

[13] Bruner KM, Wang Z, Simonetti FR, Bender AM, Kwon KJ, Sengupta S, Fray EJ, Beg SA, Antar AAR, Jenike KM (2019). A quantitative approach for measuring the reservoir of latent HIV-1 proviruses. Nature.

[14] Berkhout B (1996). Structure and function of the human immunodeficiency virus leader RNA. Prog Nucleic Acid Res Mol Biol.

[15] Graveley BR, Gilmartin GM (1996). A common mechanism for the enhancement of mRNA 3ʹ processing by U3 sequences in two distantly related lentiviruses. J Virol.

[16] Klasens BIF, Das AT, Berkhout B (1998). Inhibition of polyadenylation by stable RNA secondary structure. Nucleic Acids Res.

[17] Klasens BIF, Thiesen M, Virtanen A, Berkhout B (1999). The ability of the HIV-1 AAUAAA signal to bind polyadenylation factors is controlled by local RNA structure. Nucleic Acids Res.

[18] Ashe MP, Griffin P, James W, Proudfoot NJ (1995). Poly(A) site selection in the HIV-1 provirus: inhibition of promoter-proximal polyadenylation by the downstream major splice donor site. Genes Dev.

[19] Ashe MP, Pearson LH, Proudfoot NJ (1997). The HIV-1 5ʹ LTR poly(A) site is inactivated by U1 snRNP interaction with the downstream major splice donor site. EMBO J.

[20] Ashe MP, Furger A, Proudfoot NJ (2000). Stem-loop 1 of the U1 snRNP plays a critical role in the suppression of HIV-1 polyadenylation. RNA.

[21] Takata MA, Soll SJ, Emery A, Blanco-Melo D, Swanstrom R, Bieniasz PD (2018). Global synonymous mutagenesis identifies cis-acting RNA elements that regulate HIV-1 splicing and replication. PLoS Pathog.

[22] Smyth RP, Smith MR, Jousset AC, Despons L, Laumond G, Decoville T, Cattenoz P, Moog C, Jossinet F, Mougel M (2018). In cell mutational interference mapping experiment (in cell MIME) identifies the 5ʹ polyadenylation signal as a dual regulator of HIV-1 genomic RNA production and packaging. Nucleic Acids Res.

[23] Gilmartin GM, Fleming ES, Oetjen J, Graveley BR (1995). CPSF recognition of an HIV-1 mRNA 3ʹ-processing enhancer: multiple sequence contacts involved in poly(A) site definition. Genes Dev.

[24] Berkhout B (2000). Multiple biological roles associated with the repeat (R) region of the HIV-1 RNA genome. Adv Pharmacol.

[25] Das AT, Klaver B, Berkhout B (1999). A hairpin structure in the R region of the human immunodeficiency virus type 1 RNA genome is instrumental in polyadenylation site selection. J Virol.

[26] Das AT, Klaver B, Klasens BIF, van Wamel JLB, Berkhout B (1997). A conserved hairpin motif in the R-U5 region of the human immunodeficiency virus type 1 RNA genome is essential for replication. J Virol.

[27] Berkhout B, Klaver B, Das AT (1995). A conserved hairpin structure predicted for the poly(A) signal of human and simian immunodeficiency viruses. Virology.

[28] Harwig A, Jongejan A, van Kampen AH, Berkhout B, Das AT (2016). Tat-dependent production of an HIV-1 TAR-encoded miRNA-like small RNA. Nucleic Acids Res.

[29] Ouellet DL, Vigneault-Edwards J, Letourneau K, Gobeil LA, Plante I, Burnett JC, Rossi JJ, Provost P (2013). Regulation of host gene expression by HIV-1 TAR microRNAs. Retrovirology.

[30] Abbink TE, Berkhout B (2008). RNA structure modulates splicing efficiency at the human immunodeficiency virus type I major splice donor. J Virol.

[31] Pollom E, Dang KK, Potter EL, Gorelick RJ, Burch CL, Weeks KM, Swanstrom R (2013). Comparison of SIV and HIV-1 genomic RNA structures reveals impact of sequence evolution on conserved and non-conserved structural motifs. PLoS Pathog.

[32] Purcell DFJ, Martin MA (1993). Alternative splicing of human immunodeficiency virus type 1 mRNA modulates viral protein expression, replication, and infectivity. J Virol.

[33] Schwartz S, Felber BK, Fenyo EM, Pavlakis GN (1990). Env and Vpu proteins of human immunodeficiency virus type 1 are produced from multiple bicistronic mRNAs. J Virol.

[34] Kaiser P, Joshi SK, Kim P, Li P, Liu H, Rice AP, Wong JK, Yukl SA (2017). Assays for precise quantification of total (including short) and elongated HIV-1 transcripts. J Virol Methods.

[35] Yukl SA, Kaiser P, Kim P, Telwatte S, Joshi SK, Vu M, Lampiris H, Wong JK (2018). HIV latency in isolated patient CD4(+) T cells may be due to blocks in HIV transcriptional elongation, completion, and splicing. Sci Transl Med..

[36] Bohnlein S, Hauber J, Cullen BR (1989). Identification of a U5-specific sequence required for efficient polyadenylation within the human immunodeficiency virus long terminal repeat. J Virol.

[37] Beerens N, Groot F, Berkhout B (2000). Stabilization of the U5-leader stem in the HIV-1 RNA genome affects initiation and elongation of reverse transcription. Nucleic Acids Res.

